# Supplementary material for: Forecasting CO2 emissions of fuel vehicles for an ecological world using ensemble learning, machine learning, and deep learning models
Source: PeerJ Comput Sci. 2024 Aug 7;10:e2234. doi: 10.7717/peerj-cs.2234 (PMC11323052; doi:10.7717/peerj-cs.2234)
Supplement: Table A1 [file peerj-cs-10-2234-s002.docx]

**Table A1.** Pairwise correlation scores between all variables

|  | **X1** | **X2** | **X3** | **X4** | **X5** | **X6** | **X7** | **X8** | **X9** | **X10** | **X11** | **X12** | **X13** | **X14** | **X15** | **X16** | **X17** | **X18** | **X19** | **X20** | **X21** | **X22** | **X23** | **X24** | **Y** |
| --- | --- | --- | --- | --- | --- | --- | --- | --- | --- | --- | --- | --- | --- | --- | --- | --- | --- | --- | --- | --- | --- | --- | --- | --- | --- |
| **X1** | **1.00** | 0.93 | 0.83 | 0.76 | 0.82 | -0.76 | -0.07 | 0.16 | 0.19 | -0.24 | -0.02 | 0.29 | 0.00 | -0.20 | -0.19 | 0.19 | 0.13 | -0.23 | 0.11 | 0.04 | -0.19 | 0.01 | -0.01 | 0.25 | 0.85 |
| **X2** | 0.93 | **1.00** | 0.80 | 0.72 | 0.78 | -0.72 | -0.05 | 0.12 | 0.28 | -0.31 | 0.04 | 0.21 | 0.04 | -0.20 | -0.19 | 0.31 | 0.18 | -0.29 | 0.12 | 0.07 | -0.12 | -0.03 | 0.04 | 0.16 | 0.83 |
| **X3** | 0.83 | 0.80 | **1.00** | 0.95 | 0.99 | -0.93 | -0.11 | 0.45 | 0.16 | -0.32 | -0.03 | 0.31 | 0.05 | -0.33 | -0.16 | 0.20 | 0.13 | -0.24 | 0.12 | 0.05 | -0.21 | 0.09 | -0.09 | 0.31 | 0.92 |
| **X4** | 0.76 | 0.72 | 0.95 | **1.00** | 0.98 | -0.89 | -0.12 | 0.48 | 0.08 | -0.26 | -0.03 | 0.31 | 0.03 | -0.25 | -0.19 | 0.17 | 0.09 | -0.16 | 0.07 | 0.04 | -0.26 | 0.18 | -0.20 | 0.41 | 0.88 |
| **X5** | 0.82 | 0.78 | 0.99 | 0.98 | **1.00** | -0.93 | -0.12 | 0.47 | 0.14 | -0.30 | -0.03 | 0.31 | 0.04 | -0.31 | -0.18 | 0.20 | 0.12 | -0.21 | 0.10 | 0.04 | -0.23 | 0.12 | -0.13 | 0.35 | 0.92 |
| **X6** | -0.76 | -0.72 | -0.93 | -0.89 | -0.93 | **1.00** | 0.12 | -0.32 | -0.21 | 0.31 | 0.05 | -0.30 | -0.10 | 0.40 | 0.16 | -0.15 | -0.10 | 0.28 | -0.17 | -0.09 | 0.21 | -0.16 | 0.16 | -0.30 | -0.91 |
| **X7** | -0.07 | -0.05 | -0.11 | -0.12 | -0.12 | 0.12 | **1.00** | -0.04 | -0.14 | -0.15 | -0.03 | 0.07 | 0.01 | -0.05 | -0.04 | -0.02 | -0.03 | 0.01 | 0.02 | -0.03 | -0.05 | 0.01 | -0.04 | 0.11 | -0.03 |
| **X8** | 0.16 | 0.12 | 0.45 | 0.48 | 0.47 | -0.32 | -0.04 | **1.00** | -0.20 | -0.23 | -0.06 | 0.22 | -0.05 | -0.07 | -0.09 | -0.03 | -0.04 | 0.05 | 0.00 | -0.06 | -0.12 | 0.04 | -0.07 | 0.21 | 0.10 |
| **X9** | 0.19 | 0.28 | 0.16 | 0.08 | 0.14 | -0.21 | -0.14 | -0.20 | **1.00** | -0.86 | 0.23 | -0.20 | 0.11 | -0.18 | 0.04 | 0.13 | 0.09 | -0.59 | 0.39 | 0.28 | 0.27 | -0.20 | 0.13 | -0.28 | 0.23 |
| **X10** | -0.24 | -0.31 | -0.32 | -0.26 | -0.30 | 0.31 | -0.15 | -0.23 | -0.86 | **1.00** | -0.19 | 0.08 | -0.09 | 0.22 | 0.02 | -0.11 | -0.06 | 0.56 | -0.39 | -0.25 | -0.20 | 0.18 | -0.08 | 0.16 | -0.26 |
| **X11** | -0.02 | 0.04 | -0.03 | -0.03 | -0.03 | 0.05 | -0.03 | -0.06 | 0.23 | -0.19 | **1.00** | -0.18 | -0.27 | -0.09 | -0.14 | 0.18 | 0.01 | -0.15 | -0.06 | 0.35 | 0.15 | -0.17 | 0.10 | -0.11 | 0.00 |
| **X12** | 0.29 | 0.21 | 0.31 | 0.31 | 0.31 | -0.30 | 0.07 | 0.22 | -0.20 | 0.08 | -0.18 | **1.00** | -0.50 | -0.17 | -0.25 | 0.01 | -0.09 | -0.10 | 0.17 | -0.09 | -0.21 | 0.17 | -0.16 | 0.29 | 0.27 |
| **X13** | 0.00 | 0.04 | 0.05 | 0.03 | 0.04 | -0.10 | 0.01 | -0.05 | 0.11 | -0.09 | -0.27 | -0.50 | **1.00** | -0.25 | -0.37 | -0.09 | 0.15 | -0.04 | 0.07 | -0.14 | -0.04 | 0.06 | 0.02 | -0.06 | 0.07 |
| **X14** | -0.20 | -0.20 | -0.33 | -0.25 | -0.31 | 0.40 | -0.05 | -0.07 | -0.18 | 0.22 | -0.09 | -0.17 | -0.25 | **1.00** | -0.13 | -0.03 | -0.04 | 0.24 | -0.18 | -0.07 | -0.07 | 0.04 | 0.09 | -0.09 | -0.33 |
| **X15** | -0.19 | -0.19 | -0.16 | -0.19 | -0.18 | 0.16 | -0.04 | -0.09 | 0.04 | 0.02 | -0.14 | -0.25 | -0.37 | -0.13 | **1.00** | -0.01 | -0.08 | 0.12 | -0.12 | 0.07 | 0.24 | -0.18 | 0.02 | -0.11 | -0.17 |
| **X16** | 0.19 | 0.31 | 0.20 | 0.17 | 0.20 | -0.15 | -0.02 | -0.03 | 0.13 | -0.11 | 0.18 | 0.01 | -0.09 | -0.03 | -0.01 | **1.00** | -0.02 | -0.10 | -0.10 | -0.03 | 0.00 | -0.06 | 0.09 | -0.04 | 0.23 |
| **X17** | 0.13 | 0.18 | 0.13 | 0.09 | 0.12 | -0.10 | -0.03 | -0.04 | 0.09 | -0.06 | 0.01 | -0.09 | 0.15 | -0.04 | -0.08 | -0.02 | **1.00** | -0.18 | -0.18 | -0.05 | -0.01 | -0.01 | 0.05 | -0.05 | 0.15 |
| **X18** | -0.23 | -0.29 | -0.24 | -0.16 | -0.21 | 0.28 | 0.01 | 0.05 | -0.59 | 0.56 | -0.15 | -0.10 | -0.04 | 0.24 | 0.12 | -0.10 | -0.18 | **1.00** | -0.80 | -0.22 | -0.08 | -0.02 | -0.08 | 0.24 | -0.26 |
| **X19** | 0.11 | 0.12 | 0.12 | 0.07 | 0.10 | -0.17 | 0.02 | 0.00 | 0.39 | -0.39 | -0.06 | 0.17 | 0.07 | -0.18 | -0.12 | -0.10 | -0.18 | -0.80 | **1.00** | -0.23 | 0.03 | 0.07 | 0.02 | -0.17 | 0.12 |
| **X20** | 0.04 | 0.07 | 0.05 | 0.04 | 0.04 | -0.09 | -0.03 | -0.06 | 0.28 | -0.25 | 0.35 | -0.09 | -0.14 | -0.07 | 0.07 | -0.03 | -0.05 | -0.22 | -0.23 | **1.00** | 0.10 | -0.07 | 0.04 | -0.09 | 0.07 |
| **X21** | -0.19 | -0.12 | -0.21 | -0.26 | -0.23 | 0.21 | -0.05 | -0.12 | 0.27 | -0.20 | 0.15 | -0.21 | -0.04 | -0.07 | 0.24 | 0.00 | -0.01 | -0.08 | 0.03 | 0.10 | **1.00** | -0.37 | -0.43 | -0.22 | -0.22 |
| **X22** | 0.01 | -0.03 | 0.09 | 0.18 | 0.12 | -0.16 | 0.01 | 0.04 | -0.20 | 0.18 | -0.17 | 0.17 | 0.06 | 0.04 | -0.18 | -0.06 | -0.01 | -0.02 | 0.07 | -0.07 | -0.37 | **1.00** | -0.45 | -0.22 | 0.12 |
| **X23** | -0.01 | 0.04 | -0.09 | -0.20 | -0.13 | 0.16 | -0.04 | -0.07 | 0.13 | -0.08 | 0.10 | -0.16 | 0.02 | 0.09 | 0.02 | 0.09 | 0.05 | -0.08 | 0.02 | 0.04 | -0.43 | -0.45 | **1.00** | -0.26 | -0.12 |
| **X24** | 0.25 | 0.16 | 0.31 | 0.41 | 0.35 | -0.30 | 0.11 | 0.21 | -0.28 | 0.16 | -0.11 | 0.29 | -0.06 | -0.09 | -0.11 | -0.04 | -0.05 | 0.24 | -0.17 | -0.09 | -0.22 | -0.22 | -0.26 | **1.00** | 0.31 |
| **Y** | 0.85 | 0.83 | 0.92 | 0.88 | 0.92 | -0.91 | -0.03 | 0.10 | 0.23 | -0.26 | 0.00 | 0.27 | 0.07 | -0.33 | -0.17 | 0.23 | 0.15 | -0.26 | 0.12 | 0.07 | -0.22 | 0.12 | -0.12 | 0.31 | **1.00** |

X1: Engine_Size

X2: Cylinders

X3: Fuel_Consumption_City

X4: Fuel_Consumption_Hwy

X5: Fuel_Consumption_Comb

X6: Fuel_Consumption_Comb_MPG

X7: Fuel_Type_Diesel

X8: Fuel_Type_Ethanol

X9: Fuel_Type_Premium_Gasoline

X10: Fuel_Type_Regular_Gasoline

X11: Transmission_Automated Manual

X12: Transmission_Automatic

X13: Transmission_Automatic_Selective

X14: Transmission_CVT

X15: Transmission_Manual

X16: Make_Type_Brand_High_Performance

X17: Make_Type_Brand_Luxury

X18: Make_Type_Brand_Mainstream

X19: Make_Type_Brand_Premium

X20: Make_Type_Brand_Premium_Plus

X21: Vehicle_Class_Type_Compact

X22: Vehicle_Class_Type_SUV

X23: Vehicle_Class_Type_Sedan

X24: Vehicle_Class_Type_Truck

Y: CO2_Emissions
